# Localization of *Mycobacterium tuberculosis* topoisomerase I C-terminal sequence motif required for inhibition by endogenous toxin MazF4

**DOI:** 10.3389/fmicb.2022.1032320

**Published:** 2022-12-05

**Authors:** Pamela K. Garcia, Rosemarie Martinez Borrero, Thirunavukkarasu Annamalai, Esnel Diaz, Steve Balarezo, Purushottam B. Tiwari, Yuk-Ching Tse-Dinh

**Affiliations:** ^1^Department of Chemistry and Biochemistry, Florida International University, Miami, FL, United States; ^2^Biomolecular Sciences Institute, Florida International University, Miami, FL, United States; ^3^Department of Oncology, Georgetown University, Washington, DC, United States

**Keywords:** topoisomerase, tuberculosis, MazF toxin, TopA, mycobacteria

## Abstract

Only about half the multi-drug resistant tuberculosis (MDR-TB) cases are successfully cured. Thus, there is an urgent need of new TB treatment against a novel target. *Mycobacterium tuberculosis* (*Mtb*) topoisomerase I (TopA) is the only type IA topoisomerase in this organism and has been validated as an essential target for TB drug discovery. Toxin-antitoxin (TA) systems participate as gene regulators within bacteria. The TA systems contribute to the long-term dormancy of *Mtb* within the host-cell environment. *Mtb*’s toxin MazF4 (Rv1495) that is part of the MazEF4 TA system has been shown to have dual activities as endoribonuclease and topoisomerase I inhibitor. We have developed a complementary assay using an *Escherichia coli* strain with temperature-sensitive *topA* mutation to provide new insights into the MazF4 action. The assay showed that *E. coli* is not sensitive to the endoribonuclease activity of *Mtb* MazF4 but became vulnerable to MazF4 growth inhibition when recombinant *Mtb* TopA relaxation activity is required for growth. Results from the complementation by *Mtb* TopA mutants with C-terminal deletions showed that the lysine-rich C-terminal tail is required for interaction with MazF4. Site-directed mutagenesis is utilized to identify two lysine residues within a conserved motif in this C-terminal tail that are critical for MazF4 inhibition. We performed molecular dynamics simulations to predict the *Mtb* TopA-MazF4 complex. Our simulation results show that the complex is stabilized by hydrogen bonds and electrostatic interactions established by residues in the TopA C-terminal tail including the two conserved lysines. The mechanism of *Mtb* TopA inhibition by MazF4 could be useful for the discovery of novel inhibitors against a new antibacterial target in pathogenic mycobacteria for treatment of both TB and diseases caused by the non-tuberculosis mycobacteria (NTM).

## Introduction

*Mycobacterium tuberculosis* *(Mtb)* is the microorganism that causes Tuberculosis (TB), a leading infectious disease in the world with 10.0 million individuals experiencing active-TB, while 1.5 million succumbed in 2020 (Dean et al., 2022). The pathogen’s ability to remain dormant (also known as latent-TB) by eluding the host’s immune system, synergy between TB and AIDS, and active TB’s long 6-month therapy requirement have attributed to TB’s persistence (Kwan and Ernst, 2011; World Health Organization, 2020; Seki et al., 2021). The growing threat of drug resistance in *Mtb* poses challenge for effective treatments outcome (Kempker et al., 2015; Dean et al., 2022). As antimicrobial resistance represents a global public health threat (Boucher, 2020, eClinicalMedicine, 2021); treatments that can target TB at its latent as well as active stages are needed.

Topoisomerases are enzymes present in all domains of life (Bush et al., 2015; Garnier et al., 2021). The requirement of topoisomerases is due to its role in removing topological barriers that arise during important genomic processes, including transcription, replication, and recombination (Vos et al., 2011; Pommier et al., 2016; Brochu et al., 2020; McKie et al., 2021). Topoisomerases are classified as type I or type II topoisomerases according to the number of strands that they cleave during catalysis. Type I topoisomerases are further subclassified according to their sequence/structure homology and mechanism of actions (Baker et al., 2009). Bacterial topoisomerase I belongs to the type IA subfamily (Garnier et al., 2018; Dasgupta et al., 2020). The genome sequencing of *Mtb* confirmed that this organism encodes topoisomerase I (TopA) as the only type I topoisomerase along with gyrase as the only type II topoisomerase. Gyrase is well known as an important target for fluoroquinolones (Bush et al., 2020) including moxifloxacin used currently for treatment of drug resistant TB (World Health Organization, 2022). Genetic studies using saturation mutagenesis (Sassetti et al., 2003) and conditional knockdown (Ahmed et al., 2014; Ravishankar et al., 2015) have shown that *Mtb* TopA is essential for viability *in vitro* and *in vivo*, validating *Mtb* TopA as a target for discovery of new TB therapy (Nagaraja et al., 2017).

The high number (> 80 putative) of toxin-antitoxin (TA) systems identified in *Mtb* suggests that the TA systems may contribute to *Mtb*’s long-term dormancy and tenacity in the host (Ramage et al., 2009; Sala et al., 2014). The toxin Rv1495 (MazF4) belongs to the MazEF TA family, where MazE is the antitoxin and MazF is the toxin with sequence specific endoribonuclease activity (Zhu et al., 2006, 2008). A previous study reported that in addition to exhibiting ribonucleolytic activity, *Mtb* MazF4 interacts directly with the *Mtb* TopA and mutually inhibit each other’s enzymatic activity (Huang and He, 2010) through interaction involving the C-terminal domains (amino acid 622-934) of *Mtb* TopA. Bacterial topoisomerase I has highly conserved N-terminal domains (D1–D4) that form the active site for cleaving and rejoining of a single-strand of DNA (Baker et al., 2009; Dasgupta et al., 2020). The more variable C-terminal region (Diaz et al., 2022) also contributes to the relaxation of supercoiled DNA. The *Mtb* TopA C-terminal region has the same organization with other mycobacteria, consisting of four repeated Topo_C_Rpt domains (D5-D8) that can bind ssDNA (Tan et al., 2016; Cao et al., 2020), and a lysine-rich C-terminal tail ~ 20 amino acids in length that also plays a role in the *Mtb* TopA relaxation activity (Ahmed et al., 2013). The inhibition of *Mtb* TopA by MazF4 could potentially serve as a model for novel small molecule antibacterial compounds selective for pathogenic mycobacteria. Such antibacterial compounds may have advantage over broad-spectrum antibacterial compounds targeting TopA active site in avoiding harmful effects on the human microbiome. We report here results that further characterize the selective inhibition of *Mtb* TopA catalytic activity by MazF4, and determination of the binding site of *Mtb* MazF4 in the TopA C-terminal region.

## Materials and methods

### Cloning of *Mtb* TopA, MazF4 and *Escherichia coli* TopA

Cloning of *Mtb* TopA coding sequence in strain H37Rv into pLIC-HK vector (Corn et al., 2005) to generate plasmid pLIC-MTOP (with T7-promoter, N-terminal His-tag and kanamycin selection) has been described (Annamalai et al., 2009). The cloning of *E. coli* TopA coding sequence into the same position of pLIC-HK vector has also been reported previously (Cheng et al., 2008). The MazF4 (Rv1495) toxin gene amplified from H37Rv genomic DNA (from ATCC) was cloned using the Gibson assembly cloning procedures (Gibson, 2011) in the plasmid pBAD/Thio (from Invitrogen) using the NEB Gibson Assembly Master Mix to generate plasmid pBAD-Rv1495 (with BAD promoter, N-terminal thioredoxin tag and ampicillin resistance). Primers used in the cloning are listed in Supplementary Table S1. The clones were isolated as transformants of *Escherichia coli* NEB^®^ 5-alpha competent cells (from New England BioLabs).

### Random and site-directed mutagenesis of *Mtb* TopA

A library of mutant pLIC-MTOP plasmid with random mutations (Muteeb and Sen, 2010) in the TopA coding region was generated by first passing the plasmid through *E. coli* XL1-Red strain with *mutS*, *mutD*, and *mutT* mutations (from Agilent). The pLIC-MTOP plasmid extracted from XL1-Red was amplified in *E. coli* XL1-Blue. The coding region for TopA was excised with restriction enzymes BamHI and NcoI and ligated to the vector backbone generated from digestion of the original plasmid. The mutant pLIC-MTOP plasmid library was obtained from transformation of XL1-Blue with the ligation product.

pLIC-MTOP plasmids that express truncated version of *Mtb* TopA terminating after residue 840 or 910 were made by assembly cloning of vector and insert fragments generated with primers listed in Supplementary Table S1. Alanine substitutions of specific lysine residues in the ~ 20 a.a. C-terminal tail of *Mtb* TopA were later introduced into pLIC-MTOP plasmid using the NEB HiFi mutagenesis Master Mix and primers listed in Supplementary Table S1.

### Growth complementation assay

*Escherichia coli* strain AS17 *topA17(am)* and *Tet^r^supD43,74* with temperature sensitive *topA* is not viable at 42°C (Wang et al., 2002). The growth complementation of AS17 at the non-permissive temperature by recombinant *Mtb* TopA (Narula et al., 2010) can be tested for inhibition by MazF4 toxin when co-expressed. For the growth complementation assays, AS17 transformants of pLIC-MTOP and pBAD-Rv1495 or control plasmids were grown from single colonies overnight at 30°C in LB broth (Miller’s) in presence of kanamycin (50 μg/ml) and carbenicillin (100 μg/ml). The OD600 of the overnight cultures was adjusted to 1.0 and 10-fold serial dilutions were made with LB medium. Five microliters of serially diluted cells were spotted on LB plates (with antibiotics kanamycin 50 μg/ml and carbenicillin 100 μg/ml) containing 0.2% arabinose to induce the expression of the MazF4 toxin from the BAD promoter in pBAD-Rv1495. The plates in duplicates were incubated at 30°C for 2–3 days or 42°C for 1–2 days before they were photographed. Each complementation assay was repeated 3 times to confirm that the results are similar.

### Western blot analysis

Western blot analysis using rabbit polyclonal antibodies raised against *Mtb* TopA was conducted to compare the levels of *Mtb* TopA expression in *E. coli* AS17. Overnight cultures of AS17 transformants of pLIC-MTOP grown at 30 or 42°C were resuspended in sterile water to a concentration of OD_600_ = 10. Equal volumes of resuspended cell suspension were mixed with 2×-SDS gel loading buffer and boiled for 5 min to yield whole cell lysates. Whole cell lysates corresponding to equal cell numbers for different transformants or conditions were electrophoresed on a 10% SDS gel and transferred on to a nylon membrane for western blot analysis using rabbit anti-*Mtb* TopA (polyclonal) as primary antibody (Sandhaus et al., 2016) and goat anti-rabbit-HRP (Santa Cruz Biotechnology) as secondary antibody. The C-DiGit blot scanner (LI-COR) was used to measure the chemiluminescent western blot signal. Blotted membranes were also stained (Pierce^™^ reversible stain) to confirm for equal loading and gel transfer of proteins.

### Molecular dynamics simulation

We created a full length *Mtb* TopA (MTOP-FL) structure using RaptorX (Källberg et al., 2012) and Modeller (Šali and Blundell, 1993). MazF4 dimer structure was taken from PDB entry 5XE3 (Ahn et al., 2017). At least 50 ns molecular dynamics (MD) simulations were conducted for both MTOP-FL and MazF4 using NAMD simulation package (Phillips et al., 2005). CHARMM-GUI (Lee et al., 2016) was used to create simulation input files. Protein coordinates at the end of the simulations were used to predict a complex structure between *Mtb* TopA and MazF4 using ZDOCK webserver (Pierce et al., 2011). Finally, the docked complex was simulated for 150 ns using the NAMD simulation package. VMD (Humphrey et al., 1996) was used to visualize and analyze the simulated structures as well as to generate pictures of the complex structures. GraphPad Prism was used to plot graphs.

## Results

### *Mtb* MazF4 inhibits specifically the growth complementation of *Escherichia coli topA*^*t*s^ mutation by *Mtb* TopA

**Figure 1:**
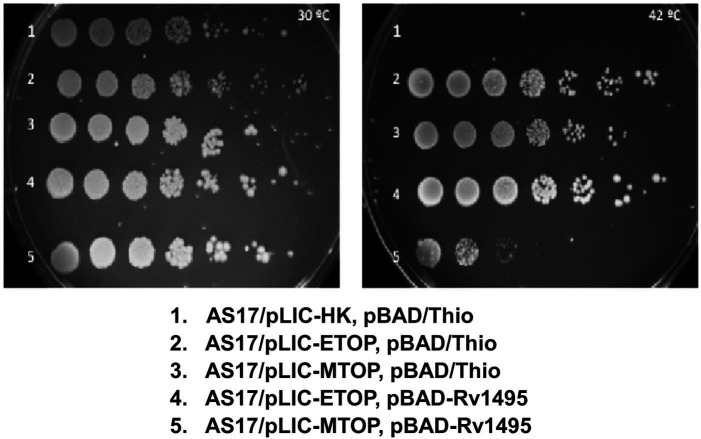
Assay for growth complementation of *Escherichia coli* AS17 with temperature sensitive *topA.* Ten-fold serial dilution of *E. coli* AS17 transformed with indicated plasmids expressing recombinant topoisomerase I (pLIC-ETOP and pLIC-MTOP), MazF4 toxin (pBAD-Rv1495) or control vectors (pLIC-HK and pBAD/Thio) were spotted on LB agar plates with antibiotics and 0.2% arabinose. The set of duplicated plates was incubated at 30°C (left) and 42°C (right).

Incubation of plates spotted with serial dilutions of overnight culture grown at 30°C (Figure 1) showed that *E. coli* strain AS17 with *topA^ts^* mutation is not viable at 42°C (Wang et al., 2002) unless complemented by recombinant *E. coli* TopA (from plasmid pLIC-ETOP) or *Mtb* TopA (from plasmid pLIC-MTOP). When an additional plasmid pBAD-Rv1495 expressing the MazF4 toxin was present in the cell, growth complementation by recombinant *E. coli* TopA was not affected, while growth of AS17 complementation by recombinant *Mtb* TopA was reduced by up to 10^3^-fold. The CFU/ml values were measured in three replicated experiments to confirm the reduction of growth complementation by *Mtb* TopA by > 200-fold due to the presence of the *Mtb* MazF4 toxin (Supplementary Table S2). The resistance of *E. coli* TopA can be explained by the absence of protein structure and sequence homology between the C-terminal domains of TopA from *E. coli* and *Mtb* (Tan et al., 2015, 2016; Dasgupta et al., 2020; Diaz et al., 2022). The lack of growth inhibition at 30°C and complementation by pLIC-ETOP provided evidence that the ribonuclease activity of MazF4 has no effect on growth of *E. coli*. *Mtb* MazF4 inhibition of AS17 growth complementation by *Mtb* TopA but not *E. coli* TopA provided support that inhibition of *Mtb* TopA growth complementation results from reduction of *in vivo* activity of *Mtb* TopA but not *E. coli* TopA. There is no T7 RNA polymerase gene in *E. coli* AS17. Western blot analysis showed that expression of recombinant *Mtb* TopA from the unknown promoter in pLIC-HK derived clone is higher at 42°C than 30°C (Supplementary Figure S1). This complementation assay provides a tool to further study the mechanism of inhibition of *Mtb* TopA by MazF4.

### Selection of mutant pLIC-MTOP clones resistant to inhibition of growth complementation by pBAD-Rv1495

**Figure 2:**
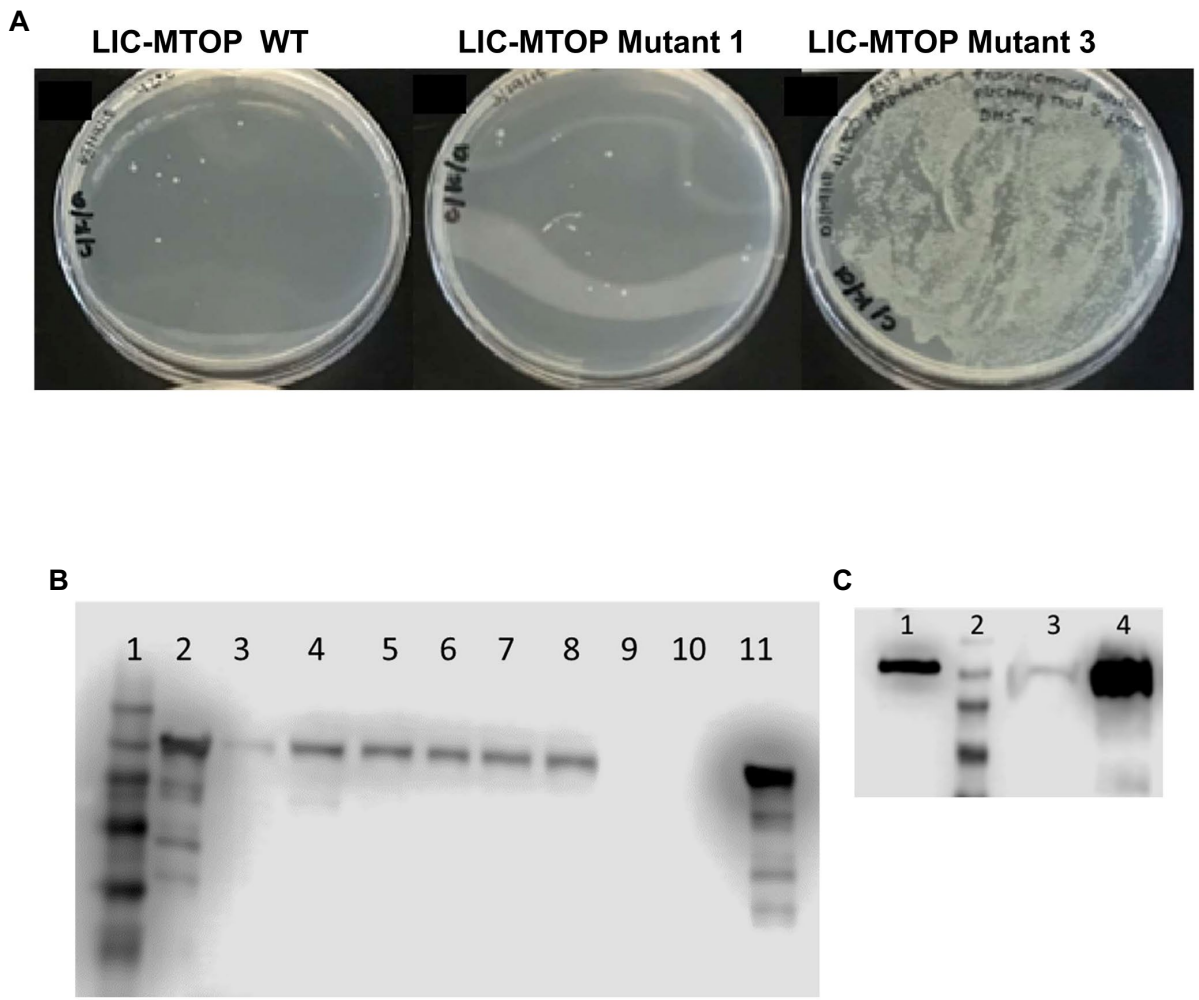
Mutant pLIC-MTOP Selected for Resistance to pBAD-Rv1495. **(A)** pLIC-MTOP plasmids were extracted from colonies of AS17/pBAD-Rv1495 transformed with mutated pLIC-MTOP library that grew at 42°C. Transformation of AS17/pBAD-Rv1495 was repeated with the extracted plasmids. Viable transformants at 42°C for the mutant-3 isolate of plasmid pLIC-MTOP showed an increase of ~ 10^4^ fold when compared to wild-type and mutant-1 isolates. **(B,C)** Western blot analysis of *Mtb* TopA expression with rabbit polyclonal antibodies raised against *Mtb* TopA. **(B)** Lane 1: prestained molecular weight standards; Lanes 2, 11: 100  ng of purified *Mtb* TopA protein; Lanes 3–8: whole cell lysate of AS17/pBAD-Rv1495 transformed with selected mutant-1 to mutant-6 isolates of pLIC-MTOP. Lanes 9, 10: whole cell lysate of AS17/pBAD-Rv1495 transformed with original pLIC-MTOP clone. **(C)** Lane 1: 1 ng of purified *Mtb* TopA protein; Lane 2: prestained molecular weight standards; Lane 3: whole cell lysate of AS17/pBAD-Rv1495 transformed with original pLIC-MTOP clone; Lane 4: whole cell lysate of AS17/pBAD-Rv1495 transformed with selected mutant-3 isolate of pLIC-MTOP. According to OD_600_ of cultures used, lane 3 corresponds to lysate from twice the number of cells compared to lysate in lane 4.

We prepared a library of mutated pLIC-MTOP plasmids by passage through the mutator strain XL1-Red. The TopA coding region was excised and ligated to the vector fragment of the original pLIC-MTOP clone. The resulting LIC-pMTOP1 library was then transformed into *E. coli* AS17 cells that have plasmid pBAD-Rv1495. Individual pLIC-MTOP plasmids were isolated from colonies that grew well at 42°C. Transformation and growth complementation assay was repeated to identify isolates of mutant pLIC-MTOP plasmid that could complement 42°C growth of AS17/pBAD-Rv1495 at much greater efficiency than the original pLIC-MTOP plasmid. Mutant-3 shown in Figure 2A supported growth of AS17/pBAD-Rv1495 better than mutant-1. However, when the TopA coding region of six selected pLIC-MTOP plasmids including mutant-1 and mutant-3 were analyzed by Sanger sequencing, no mutation could be found in the TopA coding region. When protein expression of the AS17/pBAD-Rv1495 transformants of these selected pLIC-MTOP plasmids was examined, they were found to express greatly increased level of the recombinant *Mtb* TopA protein when compared to the original pLIC-MTOP clone (Figures 2B,C). This mechanism of resistance most likely confers *in vivo* resistance to MazF4 inhibition of *Mtb* TopA effectively without cost of decreasing the relaxation activity of the *Mtb* TopA protein. Although the result did not further inform on the region of TopA that may interact directly with MazF4, it is consistent with reduction of *Mtb* TopA catalytic activity as the basis for the AS17 growth complementation inhibition by pBAD-Rv1495.

### Effect of *Mtb* TopA C-terminal truncation

**Figure 3:**
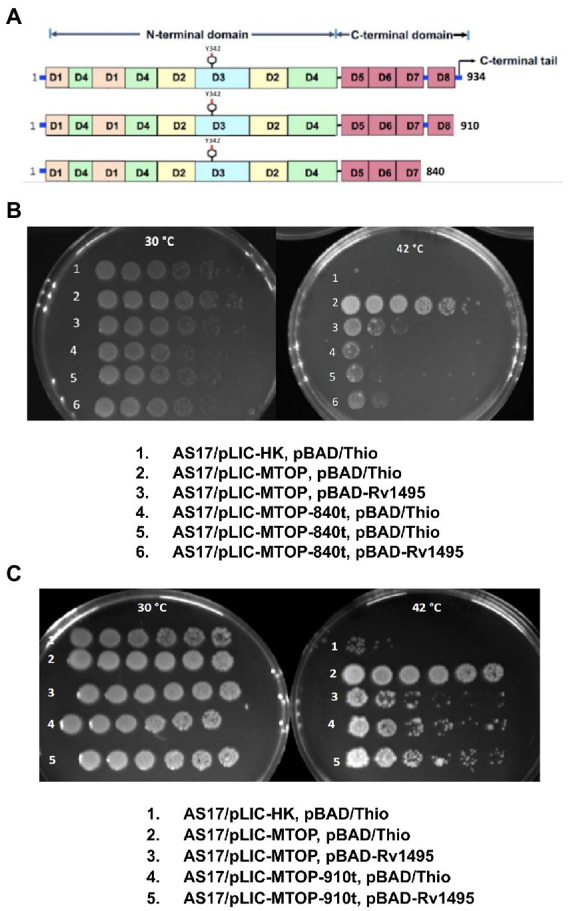
Complementation of AS17 by C-terminal truncation mutants of *Mtb* TopA. **(A)** Schematic diagram of subdomains found in full length *Mtb* TopA and mutants truncated at residue 910 and 840. Complementation of *E. coli* AS17 by pLIC-MTOP-840 t **(B)** and pLIC-MTOP-910 t **(C)** truncated mutants in the presence of pBAD/Thio or pBAD-Rv1495 was compared with complementation by pLIC-MTOP with full length *Mtb* TopA.

*Mtb* TopA (934 residues in length) is composed of two main domains: N-terminal domain and C-terminal domain (Figure 3A). The active site with Y342 for linking to DNA in the covalent intermediate is formed by the N-terminal subdomains D1–D4. The C-terminal subdomains D5-D8 consisting of repeated Topo_C_Rpt motifs (Tan et al., 2016) and the positively charged C-terminal tail that follow also interact with DNA during catalysis (Ahmed et al., 2013; Cao et al., 2020). The truncated TopA protein encoded by plasmid pLIC-MTOP-840 t lacks subdomain D8 and the C-terminal tail. Only the C-terminal tail is missing in the truncated TopA encoded in plasmid pLIC-MTOP-910 t. The growth complementation of *E. coli* AS17 at 42°C with pLIC-MTOP-840 t was weak, consistent with the reduced *in vitro* catalytic activity reported previously for *Mtb* TopA truncated at residue 840 (Cao et al., 2020), but above the background of pLIC-HK (Figure 3B). The *in vitro* catalytic activity for the mutant *Mtb* TopA truncated at residue 910 (Cao et al., 2020) and partial growth complementation by pLIC-MTOP-910 t was stronger in comparison to pLIC-MTOP-840 t (Figure 3C). The degree of complementation by both pLIC-MTOP-840 t and pLIC-MTOP-910 t was not affected by the MazF4 toxin expressed by pBAD-Rv1495. These results indicate that the presence of the TopA C-terminal tail is required for the inhibition of *Mtb* TopA activity by MazF4.

### Effect of lysine to alanine substitutions in *Mtb* TopA C-terminal tail

**Figure 4:**
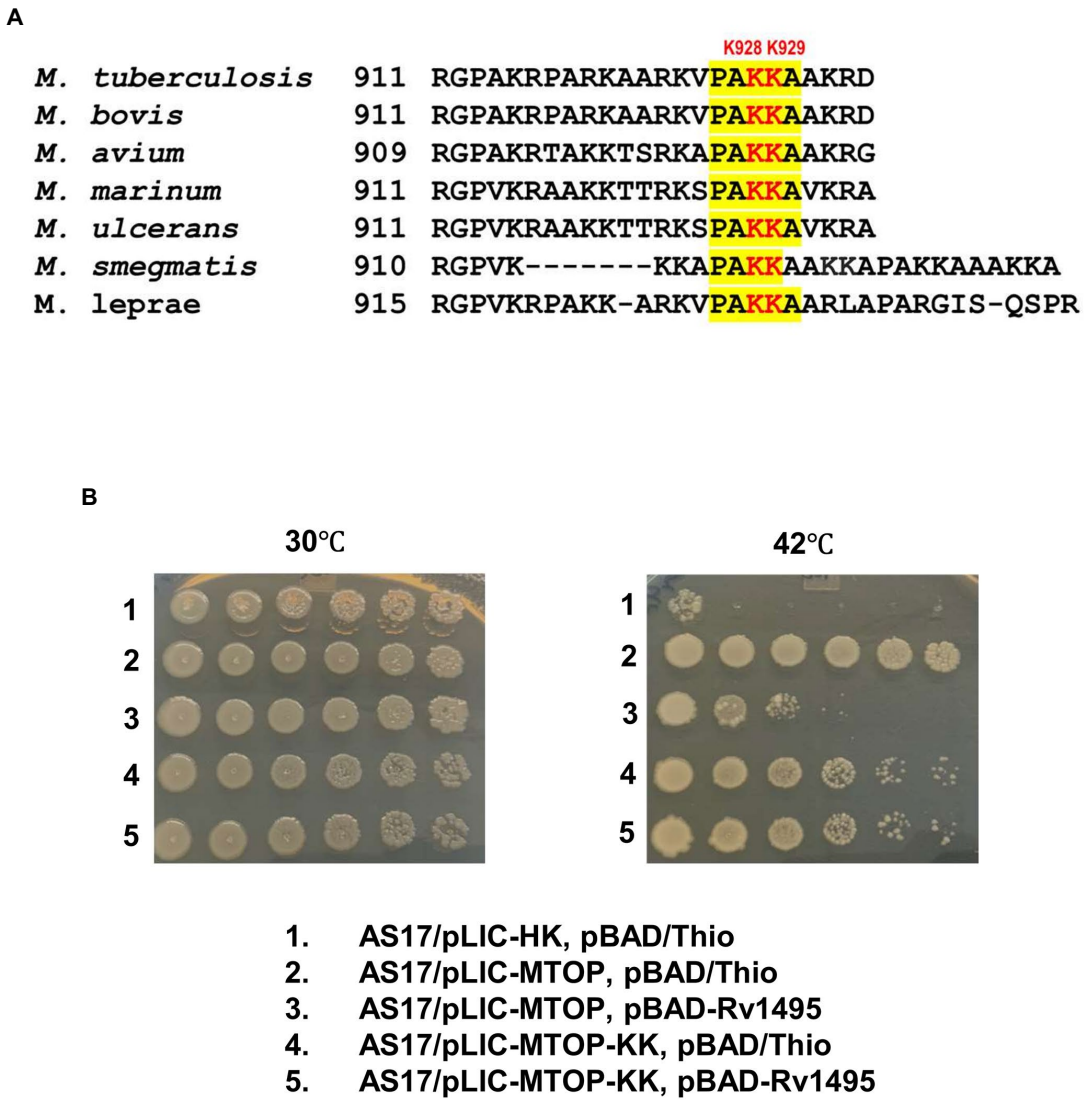
Alanine substitutions of lysines in conserved sequence present in C-terminal tail of *Mtb* TopA. **(A)** Alignment of mycobacterial TopA sequences. The conserved sequence PAKKA is highlighted. **(B)** Complementation assay of pLIC-MTOP-KK plasmid with K928A and K929A substitutions in *Mtb* TopA.

Sequence alignment of TopA from mycobacterial species shows that they share a conserved sequence of PAKKA in the lysine-rich C-terminal tail (Figure 4A). We hypothesized that a negatively charged surface of the *Mtb* MazF4 toxin (Ahn et al., 2017) interacts with the positively charged C-terminal tail of *Mtb* TopA. Site-directed alanine substitutions were made to test the potential role of lysine residues in the conserved sequence for interaction with MazF4. The plasmid pLIC-MTOP-KK encodes mutant TopA-KK with alanine substitutions at K928 and K929 within the conserved PAKKA sequence. Complementation assay (Figure 4B) showed that the TopA-KK mutant had slightly less activity as wild-type TopA for growth support of AS17 at the non-permissive temperature. The growth complementation by pLIC-MTOP-KK did not appear to be affected by the presence of pBAD-Rv1495. Measurement of CFU/ml numbers (Supplementary Table S2) at 30 and 42°C from three experimental replicates showed that there is no significant reduction in complementation (ratio of CFU at 42°C/CFU at 30°C) by pLIC-MTOP-KK when pBAD-Rv1495 is present versus pBAD/Thio (value of p from two-tailed test > 0.05). Therefore, loss of the side chains at these two lysine residues in the conserved PAKKA motif greatly reduced the inhibition of *Mtb* TopA activity by MazF4 expressed by pBAD-Rv1495.

### Molecular simulations to predict and optimize the *Mtb* TopA-MazF4 complex

**Figure 5:**
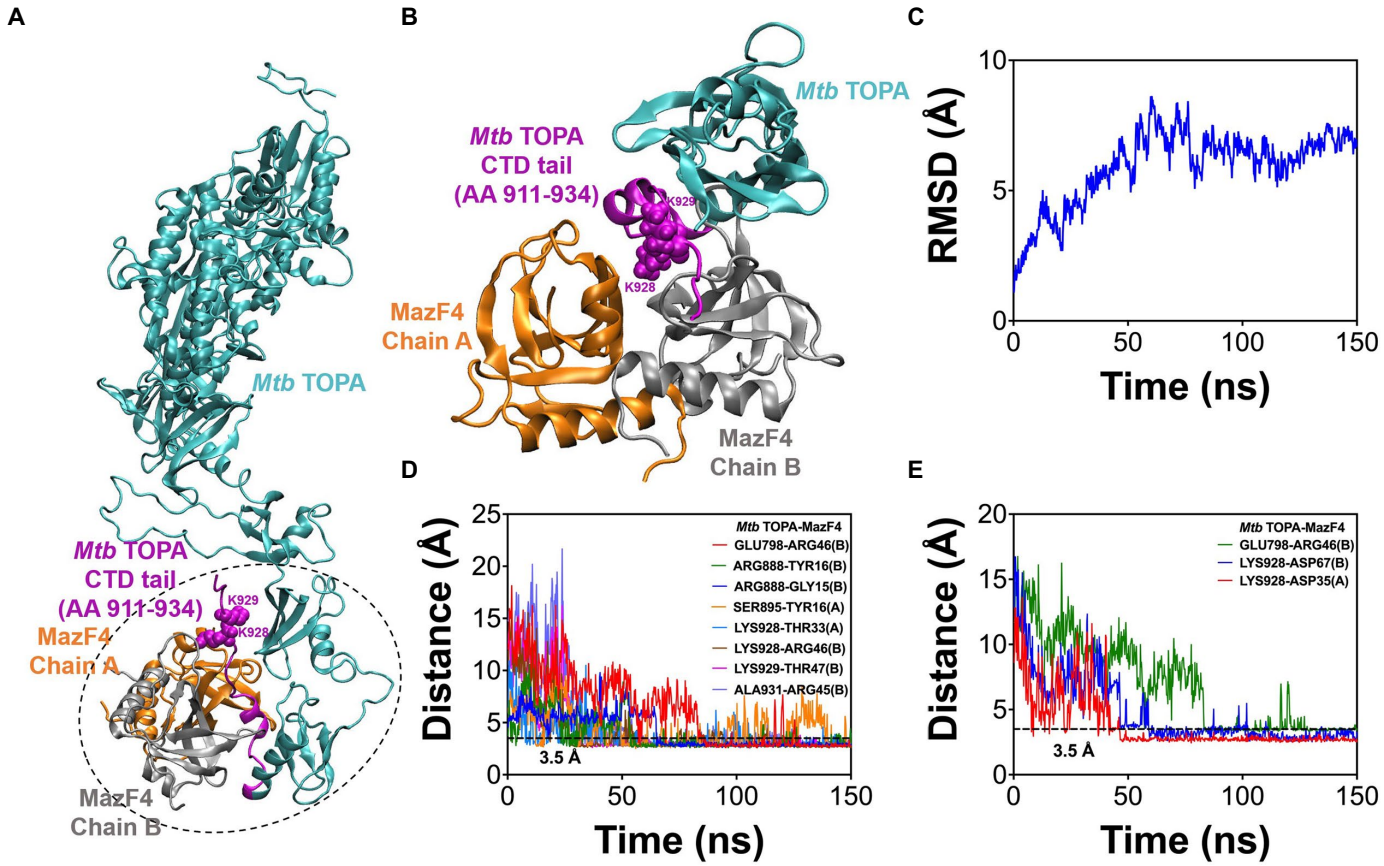
Molecular simulations of *Mtb* TopA-MazF4 complex. **(A)** Molecular docking result showing a complex formed between *Mtb* TopA and MazF4 dimer. Structures inside the dotted enclosed region were selected in molecular dynamics (MD) simulations of the complex. **(B)** *Mtb* TopA and MazF4 complex structure after 150 ns MD simulations. **(C)** RMSD measurements. **(D)** Hydrogen bonding and **(E)** salt bridge distances calculated from the 150 ns simulation trajectory.

Figure 5A shows a *Mtb* TopA-MazF4 complex predicted from molecular docking as described in the Methods section. To reduce the computational cost, we selected structure as enclosed within dotted region, which includes amino acid 786-934 portion of the *Mtb* TopA C-terminal domain (CTD) and complete MazF4 dimer. We performed MD simulations to access stability and structural integrity of the docked complex (Tiwari et al., 2016). Figure 5B shows the complex structure after 150 ns MD simulation. The complex was stable for the 150 ns simulation time as revealed by the RMSD measurements shown in Figure 5C. As shown in Figures 5D,E, several amino acid residues were found to establish the *Mtb* TopA-MazF4 complex formation through hydrogen bond and electrostatic interactions. These include K928, K929 and other amino acids in the *Mtb* TopA C-terminal tail.

## Discussion

A previous report has indicated that the binding site for MazF4 toxin is located within the 312 residue long C-terminal region of *Mtb* TopA (Huang and He, 2010). The C-terminal region of mycobacterial topoisomerase I is required for the relaxation of supercoiled DNA (Ahmed et al., 2013; Cao et al., 2020) and protein–protein interactions (Banda et al., 2017). The goal of this study is to further characterize the binding site and elucidate the mechanism of inhibition of *Mtb* TopA by MazF4.

The specific inhibition of *Mtb* TopA but not *E. coli* TopA by *Mtb* MazF4 overexpressed in the *E. coli* AS17 *topA^ts^* complementation assay provides interesting insights on the action of MazF4. It can be inferred that any specific cleavage of single-stranded RNA in *E. coli* that might occur at previously characterized sequence of U˘CGCU (˘denoting the cleavage site) by *Mtb* MazF4 (Zhu et al., 2008) did not affect the growth of *E. coli* AS17 at either 30°C or 42°C. The overexpressed *Mtb* MazF4 prevented growth of *E. coli* AS17 when the relaxation activity of recombinant *Mtb* TopA is required to complement the temperature sensitive *topA* mutation at the non-permissive temperature. This demonstrates that inhibition of the in-cell activity of *Mtb* TopA by MazF4 can result in loss of bacterial viability. The growth inhibition is specific for *Mtb* TopA as target as co-expressed *Mtb* MazF4 has no effect on the complementation by recombinant *E. coli* TopA. The finding we made that overexpression of recombinant *Mtb* TopA results in resistance to MazF4 growth inhibition provides further support that reduction of *Mtb* TopA relaxation activity is the molecular basis of the observed growth inhibition by MazF4. This mechanism of resistance to MazF4 inhibition does not affect the efficiency of complementation by *Mtb* TopA. The mutants from our attempt of targeted mutagenesis of the *Mtb* TopA coding region may not be able to produce resistant mutant *Mtb* TopA proteins without affecting the catalytic activity of *Mtb* TopA and efficiency of complementation.

Truncation of the *Mtb* TopA C-terminal domain in the LIC-MTOP-840 t (missing subdomain D9 and C-terminal tail) and LIC-MTOP-910 t (missing the 24 residue long C-terminal tail only) reduced the degree of growth complementation at 42°C because the C-terminal subdomains and C-terminal tail play important roles in interaction with DNA substrate during relaxation of supercoiled DNA (Ahmed et al., 2013; Cao et al., 2020). Nevertheless, the extent of complementation by these truncated *Mtb* TopA was not affected by the presence of *Mtb* MazF4, suggesting that the C-terminal tail is required for inhibition of *Mtb* TopA relaxation activity by MazF4.

The crystal structure of the MazEF4 complex shows a negatively charged pocket displayed by the toxin MazF4 when it is binding to the antitoxin MazE4 (Ahn et al., 2017). We hypothesize that the highly basic *Mtb* TopA C-terminal tail can be bound by the negatively charged surface of MazF4 to inhibit the TopA C-terminal domain function during catalysis. Sequence alignment of mycobacterial TopA sequences shows the presence of conserved PAKKA motif in the C-terminal tail. Alanine substitutions of the two positively charged lysine residues in *Mtb* TopA encoded in pLIC-MTOP-KK plasmid resulted in loss of nearly all the MazF4 growth inhibition in the complementation assay. Molecular dynamics simulation of *Mtb* TopA-MazF4 binding provided further support of the conserved lysine residues K928, K929 in the *Mtb* TopA C-terminal tail being involved in interaction with MazF4. In addition to electrostatic interactions, molecular simulations showed that these conserved lysine residues can also form hydrogen bonds with residues in MazF4. Other amino acid residues in the *Mtb* TopA C-terminal domain contribute to the interactions with MazF4 as well.

**Figure 6:**
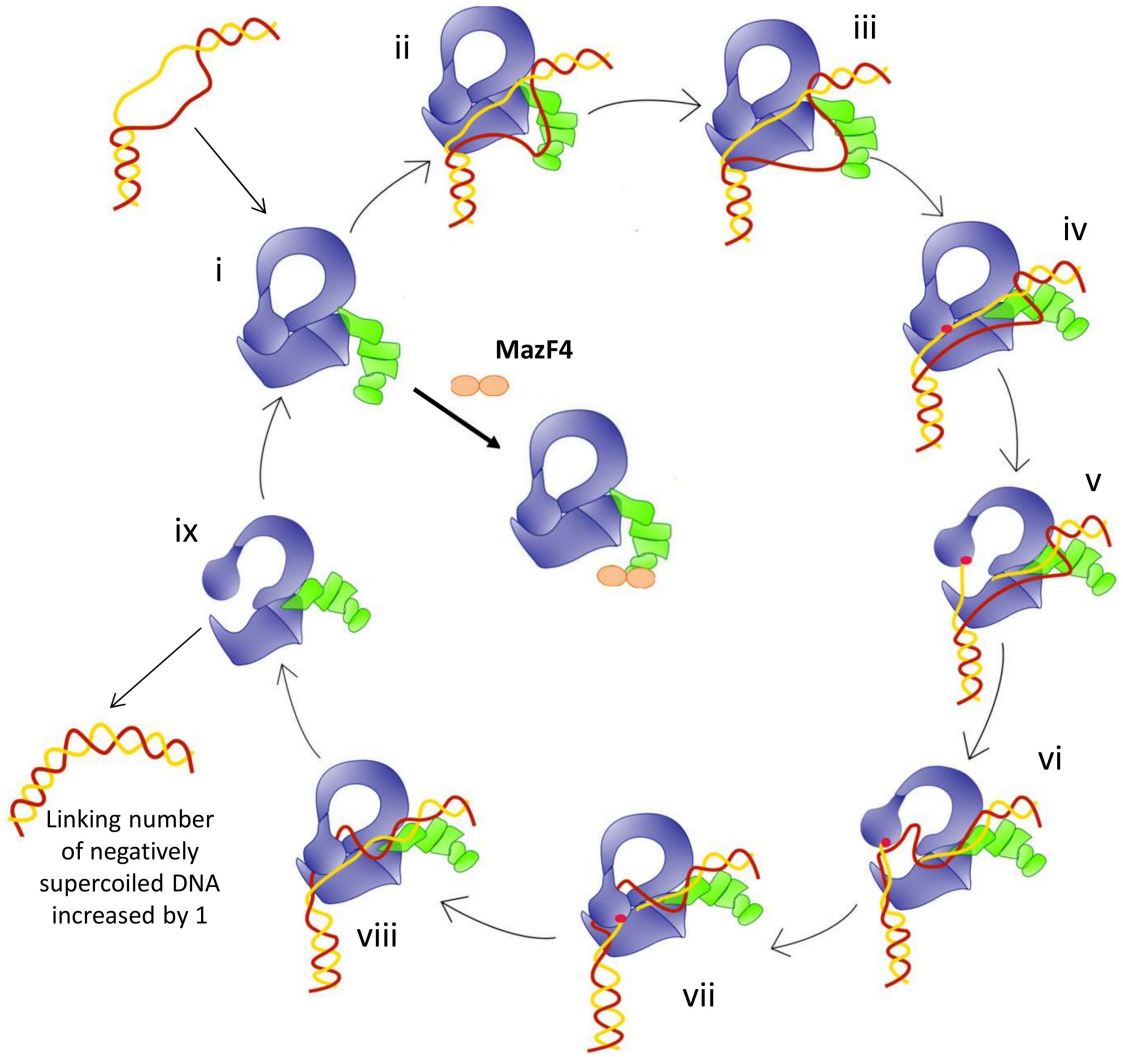
Model for the relaxation of supercoiled DNA by bacterial topoisomerase I based on the crystal structures of *E. coli* and Mycobacteria topoisomerase I. (i) Apo enzyme; (ii) C-terminal domains (green) recognizes ssDNA region in unwound DNA duplex as T-strand (red); (iii) ssDNA or G-strand (yellow) binds the N-terminal domains (blue); (iv) Active site tyrosine (red circle) becomes accessible; (v) Cleavage of the G-strand and gate opening with T-strand DNA approaching toroid hole; (vi) Passage of T-strand inside the toroid; (vii) Gate closing and trapping of T-strand; (viii) Religation of the G-strand; (ix) Gate opening and release of dsDNA. Figure reproduced from Dasgupta et al. (2020).

The model of relaxation of supercoiled DNA by bacterial TopA is shown in Figure 6. Previous biochemical studies (Ahmed et al., 2013; Cao et al., 2020) on mycobacterial TopA showed that the high affinity of the C-terminal domains and basic tail for ssDNA is important for recognition of the unwound duplex DNA substrate and promote strand passage (step ii and step v, Figure 6). The formation of a stable *Mtb* TopA-MazF4 complex is likely to inhibit the catalytic function of the *Mtb* TopA.

*Mtb* TopA is a validated target for discovery of novel TB drug leads (Godbole et al., 2015; Ravishankar et al., 2015; Nagaraja et al., 2017). Inhibitors that act at the highly conserved N-terminal domains of bacterial TopA might be more likely to be broad-spectrum in antibacterial action and could have undesirable effect on the normal human microbiota. This study has led to further understanding of the action of *Mtb* toxin MazF4. It suggests that the *in vivo* relaxation activity of *Mtb* TopA can be inhibited through specific targeting of its C-terminal tail to achieve select growth inhibition of *Mtb* and possibly related mycobacteria. We have not included in this study characterization of the *in vitro* interaction between *Mtb* TopA and MazF4. Such characterization is needed in future studies to compare the action of MazF4 with small molecule inhibitors that might also be binding at the *Mtb* TopA C-terminal tail.

## Data availability statement

The original contributions presented in the study are included in the article/Supplementary material, further inquiries can be directed to the corresponding author.

## Author contributions

Y-CT-D and TA conceived the idea and designed the study. PG, RB, ED, and PT executed the experiments. TA, PG, and PBT performed the analyses. Y-CT-D wrote the draft of the manuscript. All authors contributed to the article and approved the submitted version.

## Funding

This research was supported by the National Institute of General Medical Sciences of the National Institutes of Health under Award Number R35GM139817, R01GM054226, and Supplement GM054226-S1 (to Y-CT-D), Colciencias (Convocatoria 586 de becas en el exterior to PG). PBT acknowledges the computational resources acquired with a research restart discretionary fund from the office of the Dean for Research at Georgetown University Medical Center.

## Conflict of interest

The authors declare that the research was conducted in the absence of any commercial or financial relationships that could be construed as a potential conflict of interest.

## Publisher’s note

All claims expressed in this article are solely those of the authors and do not necessarily represent those of their affiliated organizations, or those of the publisher, the editors and the reviewers. Any product that may be evaluated in this article, or claim that may be made by its manufacturer, is not guaranteed or endorsed by the publisher.

## Supplementary material

The Supplementary material for this article can be found online at: https://www.frontiersin.org/articles/10.3389/fmicb.2022.1032320/full#supplementary-material
