# Supplementary material for: Localization of Mycobacterium tuberculosis topoisomerase I C-terminal sequence motif required for inhibition by endogenous toxin MazF4
Source: Front Microbiol. 2022 Dec 5;13:1032320. doi: 10.3389/fmicb.2022.1032320 (PMC9760754; doi:10.3389/fmicb.2022.1032320)
Supplement: Supplementary file 1 [file Data_Sheet_1.PDF]

Table S1. Oligonucleotide Primers Used in This Study

**Primers for cloning Rv1495 into pBAD/Thio**	
pBAD/Thio For	AAGGGCGAGCTTGAAGGT
pBAD/Thio Rev	AAGGGCGAGCTTGTCATC
Rv1495 For1	gacaagctcgcccttGTGAACGCGCCGTTGCGT
Rv1495 Rev1	cttcaagctcgcccttTGGCCACGGTAGCCCCAG
**Primers for generating pLIC-MTOP-840t[1]**	
Vector1 For	CCGAATTCGAGCGCCGTCG
Vector1 Rev	TTTCATGGTGATGGTGATGGTG
Insert1 For	ccatcaccatcaccatgaaaACCTGTACTTCCAATCCAATGCA
Insert1 Rev	tcgacggcgctcgaattcggATCCGTTATCCACTTCCAATG
**Primers for generating pLIC-MTOP-910t[2]**	
Vector2 For	TAATAACATTGGAAGTGGATAACGGATCCGAATTCGAG
Vector2 Rev	TGCATTGGATTGGAAGTACAGGTTTTC
Insert2 For	tgtacttccaatccaatgcaGCTGACCCGAAAACGAAGGG
Insert2 Rev	atccacttccaatgttattaCTAGGCTCGGCGATCGGC
**Primers for K928A, K929A mutagenesis[2]**	
For	GGTGCCGGCGGCCGCCGCAGCCAAGC
Rev	TTCCGGGCAGCTTTCCTG

[1]Plasmid 2O-T-MtbTOP1-840t ((Cao et al., 2020) was used as template for the insert..

[2]Plasmid pLIC-MTOP (Annamalai et al., 2009) was used as template for the insert.

References

ANNAMALAI, T., DANI, N., CHENG, B. & TSE-DINH, Y. C. 2009. Analysis of DNA relaxation and cleavage activities of recombinant Mycobacterium tuberculosis DNA topoisomerase I from a new expression and purification protocol. *BMC Biochem,* 10**,** 18.

CAO, N., TAN, K., ZUO, X., ANNAMALAI, T. & TSE-DINH, Y. C. 2020. Mechanistic insights from structure of Mycobacterium smegmatis topoisomerase I with ssDNA bound to both N- and C-terminal domains. *Nucleic Acids Res,* 48**,** 4448-4462.

Table S2. CFU/ml from plates incubated at 42degC and 30degC

CFU/ml were counted following incubation of plates at 42degC for 1 day and 30degC for 2 days. Average and standard deviation were determined for the ratio of colonies formed at 42 and 30 degC from three independent experiments.

CFU/ml	42degC	30degC	42/30 Ratio
1.AS17/pLIC-HK, pBAD/Thio	5.60E+02	1.08E+07	5.19E-05
	7.60E+02	2.84E+07	2.68E-05
	9.20E+02	1.20E+07	7.67E-05
Ave			5.18E-05
Stdev			2.50E-05
2.AS17/pLIC-MTOP, pBAD/Thio	2.20E+07	2.04E+07	1.08E+00
	1.12E+08	1.24E+08	9.03E-01
	1.04E+08	1.16E+08	8.97E-01
Ave			9.59E-01
stdev			1.03E-01
3.AS17/pLIC-MTOP, pBAD-Rv1495	2.80E+05	1.36E+08	2.06E-03
	2.00E+05	1.28E+08	1.56E-03
	2.90E+06	3.18E+08	9.12E-03
Ave			4.25E-03
stdev			4.23E-03
4.AS17/pLIC-MTOP-KK, pBAD/Thio	1.84E+07	1.00E+08	1.84E-01
	1.04E+07	2.12E+07	4.91E-01
	2.96E+06	1.25E+07	2.37E-01
Ave			3.04E-01
stdev			1.64E-01
5.AS17/pLIC-MTOP-KK, pBAD-Rv1495	2.04E+06	2.08E+07	9.81E-02
	1.20E+07	1.20E+08	1.00E-01
	1.76E+06	1.35E+07	1.30E-01
Ave			1.09E-01
stdev			0.018115

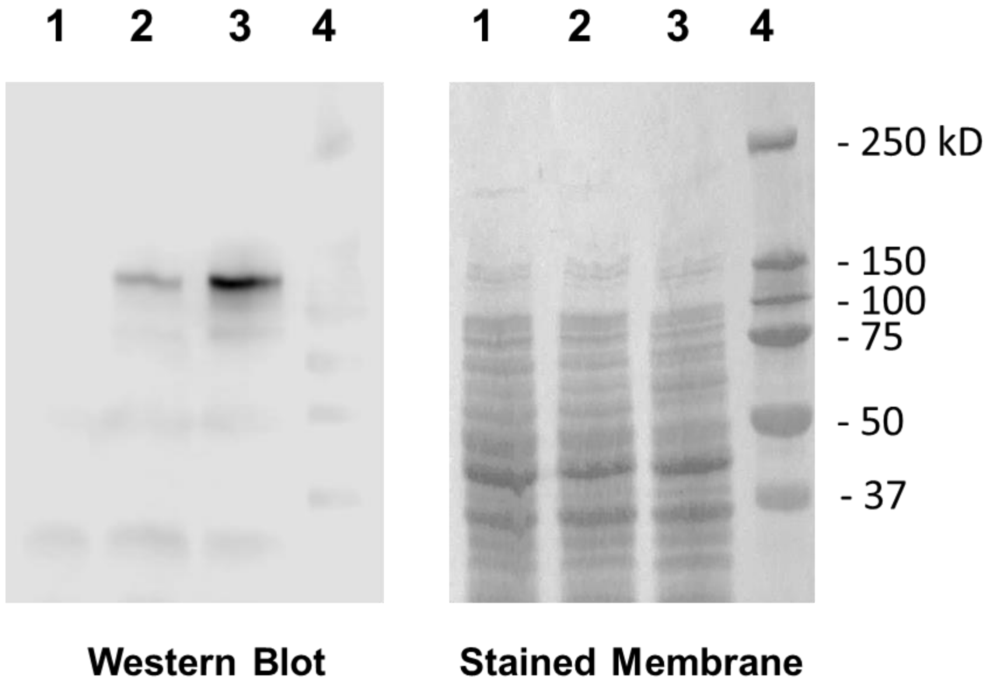

Figure S1. Expression of recombinant *Mtb* TopA from pLIC-MTOP in *E. coli* AS17. Lane 1: AS17/pLIC-HK, 30°C; Lane 2: AS17/pLIC-MTOP, 30°C; Lane 3: AS17/pLIC-MTOP, 42°C; Lane 4: molecular weight standards. Western blot was conducted using rabbit polyclonal antibodies raised against *Mtb* TopA protein. Blotted membrane was stained with Pierce^{TM} reversible stain.
